# Genome Mining of Non-Conventional Yeasts: Search and Analysis of *MAL* Clusters and Proteins

**DOI:** 10.3390/genes9070354

**Published:** 2018-07-16

**Authors:** Katrin Viigand, Kristina Põšnograjeva, Triinu Visnapuu, Tiina Alamäe

**Affiliations:** Department of Genetics, Institute of Molecular and Cell Biology, University of Tartu, Riia 23, 51010 Tartu, Estonia; katrin.viigand@ut.ee (K.V.); kristina.poshnograjeva@gmail.com (K.P.); triinu.visnapuu@ut.ee (T.V.)

**Keywords:** α-glucosidase, maltase, isomaltase, α-glucoside permease, gene cluster, protein evolution, methylotrophic yeast, *MAL*-locus

## Abstract

Genomic clustering of functionally related genes is rare in yeasts and other eukaryotes with only few examples available. Here, we summarize our data on a nontelomeric *MAL* cluster of a non-conventional methylotrophic yeast *Ogataea* (*Hansenula*) *polymorpha* containing genes for α-glucosidase MAL1, α-glucoside permease MAL2 and two hypothetical transcriptional activators. Using genome mining, we detected *MAL* clusters of varied number, position and composition in many other maltose-assimilating non-conventional yeasts from different phylogenetic groups. The highest number of *MAL* clusters was detected in *Lipomyces starkeyi* while no *MAL* clusters were found in *Schizosaccharomyces pombe* and *Blastobotrys adeninivorans*. Phylograms of α-glucosidases and α-glucoside transporters of yeasts agreed with phylogenesis of the respective yeast species. Substrate specificity of unstudied α-glucosidases was predicted from protein sequence analysis. Specific activities of *Scheffersomyces* *stipitis* α-glucosidases MAL7, MAL8, and MAL9 heterologously expressed in *Escherichia coli* confirmed the correctness of the prediction—these proteins were verified promiscuous maltase-isomaltases. α-Glucosidases of earlier diverged yeasts *L. starkeyi*, *B. adeninivorans* and *S. pombe* showed sequence relatedness with α-glucosidases of filamentous fungi and bacilli.

## 1. Introduction

Maltose utilization in yeasts has been studied mostly in *Saccharomyces* as these yeasts are commonly used in brewing. The beer wort contains 50–60% of maltose, 15–20% maltotriose, and some other sugars, including isomaltose. These oligosaccharides are transported into the yeast cell, hydrolyzed to glucose by maltases and isomaltases, and fermented to ethanol and CO_2_ [1].

*Ogataea* (formerly *Hansenula*) *polymorpha*, *Komagatella phaffii* (formerly *Pichia pastoris*), and *Candida boidinii* are the most thoroughly studied methylotrophic yeasts [2,3,4]. We showed that one of these species—*Ogataea polymorpha* (*Op*)—can grow on maltose and sucrose using maltase (an α-glucosidase) for their hydrolysis [5,6]. In following studies, we focused on genetics, biochemistry, and regulation of utilization of α-glucosidic sugars in *Op* [7,8,9,10,11,12].

In *Saccharomyces*, α-glucosidic oligosaccharides are hydrolyzed by two specialized enzymes—maltases (EC 3.2.1.20) that degrade maltose and maltotriose (both α-1,4 linked), and isomaltases (EC 3.2.1.10) that degrade α-1,6 linked substrates such as isomaltose and palatinose [13,14,15]. These specific α-glucosidases (AG_S_) of *Saccharomyces* most probably evolved from a promiscuous ancestral protein ancMALS through gene duplications and further evolution of a duplicate [13]. Interestingly, we recently showed [12] that resurrected ancMALS and the *Op* maltase MAL1 are highly similar according to substrate usage and signature amino acids potentially involved in substrate binding. As *Op* belongs to an earlier diverged lineage of the ascomycetes [16], the presence of a living ‘twin’ of the hypothetical ancMALS protein supports the hypothesis raised in [13]. The *Op* MAL1 is a promiscuous enzyme with perfect catalytic properties towards a wide range of substrates: maltose, maltotriose, maltulose, sucrose, turanose, melezitose, isomaltose, isomalto-oligosaccharides, palatinose, and α-methylglucoside (α-MG). Therefore the MAL1 of *O. polymorpha* was regarded as maltase-isomaltase rather than a maltase [12].

In *Saccharomyces cerevisiae*, the genes required for utilization of α-glucosidic sugars are clustered in subtelomeric regions of the chromosomes forming so-called *MAL* clusters or loci. Genomic clustering of functionally related genes is rare in yeasts and other eukaryotes. Yet, aside from *MAL* loci, metabolic clusters for the utilization of galactose, allantoine, and nitrate are described in yeasts and filamentous fungi [17,18,19,20]. As emphasized in [21], metabolic gene clusters confer a survival advantage to the host when coinherited. As minimum, the *MAL* cluster of *S. cerevisiae* contains genes encoding a permease, an AG (maltase or isomaltase) and a transcriptional activator of the *MAL* genes. The number and composition of *MAL* clusters, as well as properties of encoded proteins, vary between the strains of *S. cerevisiae* [22,23,24]. In addition to *Saccharomyces*, the *MAL* loci have also been described for few other yeasts such as *Scheffersomyces* (*Pichia*) *stipitis*, an efficient fermenter of lignocellulosic sugars that has at least 35 loci of functionally linked genes [21,25]. However, the properties of the proteins encoded by *S. stipitis MAL* clusters have not been studied.

Clustering of *MAL* genes has also been shown for non-conventional yeasts *Op* [10] and *Ogataea parapolymorpha* (*Opp*) [26]. In the current work, we (i) searched for *MAL* clusters from the genomes of other non-conventional yeasts; (ii) performed the phylogenetic analysis of AGs and α-glucoside transporters (AGTs) encoded by the clusters; (iii) predicted substrate specificity of AGs using protein sequence analysis; and (iv) evaluated the correctness of the prediction by enzymatic analysis of three heterologously expressed AGs of *S. stipitis*.

## 2. Materials and Methods

### 2.1. The Yeasts and the Genomes

Yeast strains and genomes analyzed in the current study are listed in Table 1. Most of the genomes were accessed through the MycoCosm portal http://genome.jgi.doe.gov/programs/fungi/index.jsf [27]. Two *Ogataea* strains were studied: *O. polymorpha* (*Op*) and *O. parapolymorpha* (*Opp*) (Table 1). The first genome of *Op* (of RB11 strain, an *odc1* derivative of CBS4732) was sequenced 15 years ago [28], but it has not yet been released to the public domain. The *Op* strains NCYC 495 (Table 1) and CBS 4732 mate, yield viable spores, and are almost identical in DNA sequence [29,30,31]. The genomes of *Opp* DL-1 [26], *Meyerozyma guillermondii* and *Lodderomyces elongisporus* [32] in MycoCosm originate from the National Center of Biolotechnology Information (NCBI). The genome of *Debaryomyces hansenii* is present as a copy from Génolevures Project in MycoCosm [33]. The genome sequence and gene predictions of *Blastobotrys* (*Arxula*) *adeninivorans* strain LS3 were obtained by Cécile Neuvéglise from MycoCosm. This genome of *B. adeninivorans* was originally sequenced by the Génolevures consortium [33]. The *Schizosaccharomyces pombe* genome in MycoCosm is a copy from www.pombase.org. *MAL* genes and clusters of *S. cerevisiae* S288C were used as a reference. *S. stipitis* CBS 6054 used in growth assay on sugars and cloning of the *AG* genes was kindly provided by Prof. A. Sibirny (Lviv, Ukraine).

### 2.2. Extraction of DNA and Protein Sequences and Analysis of Genomic Neighborhood of *AG* Genes to Detect *MAL* Clusters

Potential *MAL* genes were searched by using two different approaches. First, the Blast searches were run in GenBank (https://www.ncbi.nlm.nih.gov/genbank/) and MycoCosm websites against respective *Op* AG (MAL1) and AGT (MAL2) proteins to retrieve the genes encoding related proteins from other yeasts. Additionally, potential genes of interest were searched by their predicted function in the KOG (EuKaryotic Orthologous Groups) tab in MycoCosm webpage. Genes predicted to function in carbohydrate transport and metabolism were investigated further. The neighboring areas of the revealed genes were investigated using annotation data in the Synteny menu of MycoCosm. The *MAL* cluster was defined as a cluster comprising at least two potential *MAL* genes, with one of them encoding an AG. Due to that, not all potential *MAL* genes of studied species were covered in this study. For example, eight *MAL* clusters containing an *AG* gene were detected in *Lipomyces starkeyi* genome, but this yeast has several *AG* genes outside the clusters. pDRAW32 v1.1.107 (http://www.acaclone.com/) was used for analysis and visualization of the sequences of *D. hansenii*. Information on introns in the genes was extracted from MycoCosm and GenBank. Accession numbers and acronyms of the genes and proteins are given in Tables S1 and S2.

### 2.3. Alignment of Gene and Protein Sequences for Identity Evaluation, Construction of Phylogenetic Trees, and Defining Signature Amino Acids

Gene sequences from domains 1 and 2 (D1/D2) of large subunit ribosomal RNA (rRNA) [16] were used to build a phylogenetic tree of yeast species. As we could not find the large subunit rRNA sequences in genomic scaffolds of *Cyberlindnera fabianii* YJS4271, respective D1/D2 sequence (KY107353.1) of *C. fabianii* type strain CBS 5640 was used instead.

MEGA 7.0 package [40] was used to calculate neighbor-joining phylogenetic trees [41]. The Dayhoff model [42] was used for the protein (AGs and AGTs) phylograms, and the maximum composite likelyhood model [43] for the rRNA genes, with 1000 bootstrap replicates in both cases. The sequences were aligned using ClustalW [44] to calculate the identity values between the proteins presented in Tables S3 and S4 and to define the signature amino acids [12,13] suggested crucial for sugar binding. The genes were translated using respective alternative genetic code in case of CTG clade yeasts.

### 2.4. Heterologous Expression of AGs Encoded in the Genome of *Scheffersomyces stipitis* and Substrate Specificity Assay of the Enzymes

Three AGs: MAL7, MAL8 and MAL9 encoded by *S. stipitis MAL* loci [21] were cloned from *S. stipitis* CBS 6054 genomic DNA isolated with a PowerSoil Kit (MoBio, Carlsbad, CA, USA). Plasmid pY6 [45], kindly provided by C. Michels (New York, NY, USA), was used as a source of *S. cerevisiae* maltase gene *MAL62.* The polymerase chain reaction (PCR)-amplified genes were first inserted into pJET vector (Thermo Fisher Scientific, Waltham, MA, USA) and further cloned into pURI3-Cter vector [46]. Recombinant Pfu polymerase (Thermo Fisher Scientific, Waltham, MA, USA) was used in amplification and cloning, for primers see Tables S5 and S6. Resulting plasmids were electroporated into *Escherichia coli coli* BL21 (DE3) for heterologous expression of the AG proteins. Respective *E. coli* transformants were grown in 200 mL of lysogeny broth (LB) ampicillin (0.15 mg/mL) medium at 37 °C on a shaker to an optical density (OD) at 600 nm of ~0.5. Then 0.5 mM isopropyl β-d-1-thiogalactopyranoside (IPTG) was added, the temperature was shifted to 22 °C and bacteria were further grown for ~20 h. Cells were harvested by centrifugation (2400× *g*, 20 min), washed in maltase buffer (100 mM K-phosphate buffer with 0.1 mM ethylenediaminetetraacetic acid (EDTA), pH 6.5)), suspended in the same buffer and crude cell extracts were prepared as in [6]. The cell extracts were assayed for the hydrolysis of 1 mM PNPG (*p*-nitrophenyl-α-d-glucopyranoside) and 100 mM sucrose, maltose, α-MG and palatinose in maltase buffer at 30 °C. Initial velocity of the reaction was measured by recording *p*-nitrophenol release (in case of PNPG) or glucose release in case of other substrates [47]. The activity was normalized to protein concentration in the extracts measured using a BCA Protein Assay Kit (Thermo Fisher Scientific, Waltham, MA, USA) and expressed in micromoles of hydrolyzed substrate per min per mg of protein in the extract (µmol/mg∗min; U/mg). Hydrolysis of the above listed substrates by cell extract of *E. coli* BL21 (DE3) transformant carrying the empty pURI3-Cter vector was assayed to register possible background activity.

### 2.5. Assay of *Scheffersomyces stipitis* Growth Ability on Sugars and Evaluation of Hydrolysis of α-Glucosidic Sugars by *S. stipitis *Cell Extracts

*S. stipitis* was grown in 0.67% BD Difco Yeast Nitrogen Base (YNB) medium (Thermo Fisher Scientific, Waltham, MA, USA) without amino acids supplemented with His (50 mg/L), Trp (50 mg/L), and 0.2% of a sugar (glucose, maltose, maltotriose, maltulose, sucrose, turanose, palatinose, xylose, or α-MG) on Greiner 96-well flat-bottom transparent polystyrol microplates (Greiner Bio-One, Frickenhausen, Germany) under agitation for 20 h at 30 °C. Cells grown overnight on 0.2% glucose were used for inoculation. Optical density of the culture at 600 nm was measured at the beginning and at the end of the experiment using an Infinite M200 PRO microplate reader (Tecan Group Ltd., Männedorf, Switzerland) equipped with Tecan i-control 1.7 software.

For enzymatic assay, *S. stipitis* was grown till mid-exponential growth phase in His- and Trp-supplemented YNB medium containing 1% of either glucose, maltose, sucrose, palatinose, or xylose. Sugars were added to growth medium from filter-sterilized (pore size 0.22 µm) solutions. The cells were collected by centrifugation at 4 °C, washed twice in maltase buffer and disrupted by using glass-beads. Supernatants of disrupted cells [6] were used as crude cell extract to measure specific activity of hydrolysis of PNPG, maltose, sucrose, palatinose and α-MG as described above.

## 3. Results

### 3.1. *MAL* Genes are Clustered in *Ogataea polymorpha (Op)* and *O. parapolymorpha (Opp)*

**Figure 1:**
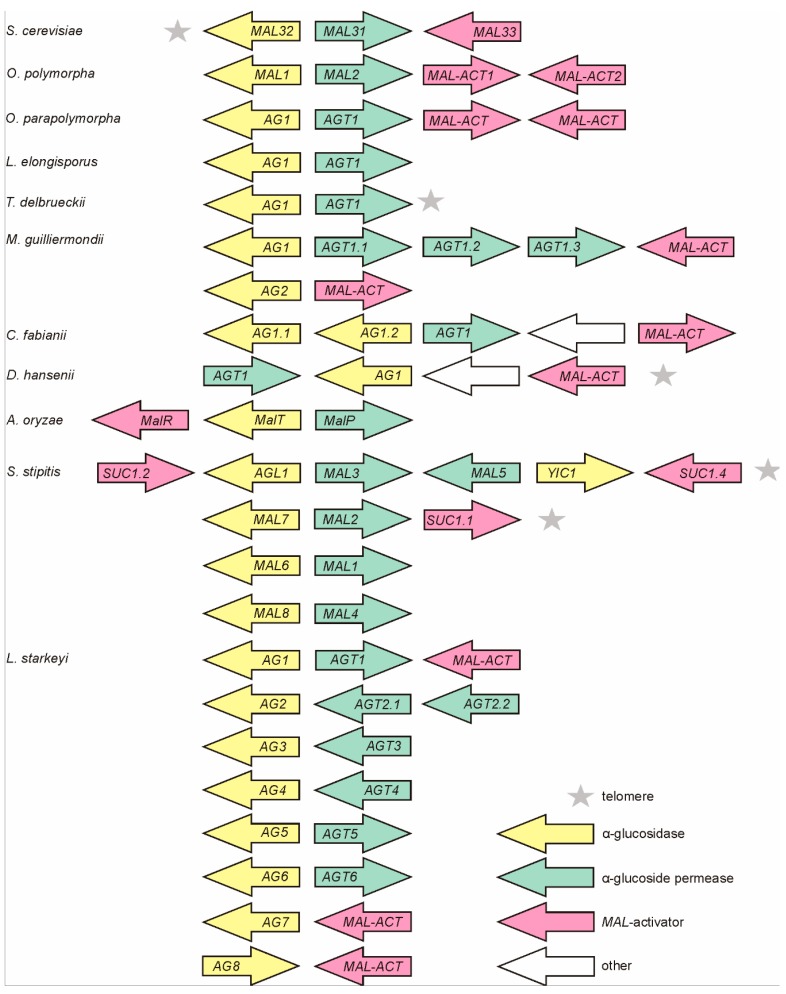
*MAL* loci and their composition in *Ogataea polymorpha* (*Op*) NCYC 495, *Ogataea parapolymorpha* (*Opp*) DL-1 and other non-conventional yeasts. Genes (potentially) encoding α-glucosidases (AG), α-glucoside transporters (AGT), and *MAL*-activators (*MAL*-ACT) were retrieved from the genomic databases (see Table 1). The *MAL* clusters of *Scheffersomyces stipitis* [21], *Aspergillus oryzae* [50], and *Saccharomyces cerevisiae* (*MAL32*—maltase; *MAL31*—α-glucoside permease; *MAL33*—*MAL*-activator) are presented for comparison. Accession numbers and annotation data of the *AG* and *AGT* genes are given in Tables S1 and S2.

Sequencing of *MAL* genes in inserts of genomic library clones of *Op* CBS 4732 [8,10] revealed a four-gene *MAL* cluster. The genes for AG (*MAL1*) and AGT (*MAL2*) were shown adjacent sharing a bi-directional promoter [10] (Figure 1) as in the case of *S. cerevisiae* [48]. The *MAL* clusters of *Op* (in chromosome 1) and *Opp* (in chromosome 7) occurred similar (see Table S7 regarding the synteny). The sequence identity of the AGTs encoded in *MAL* clusters of *Op* and *Opp* was 87% and respective value for AGs was 98%. The *MAL* clusters of *Op* and *Opp* were not subtelomeric—the distance of *Op MAL* cluster from the chromosome end was about 410,000 bp, and the respective distance for *Opp* was 380,000 bp.

Interestingly, we detected hypothetical *AGT* and *AG* genes outside of the above-mentioned *MAL* cluster (Table S8) in both *Ogataea* species. However, we considered that these genes were not required for the growth of these yeasts on α-glucosidic sugars. If either the *MAL1* or *MAL2* gene of the *MAL* locus of *Op* was disrupted, the cells lost the ability to assimilate maltose, sucrose, turanose, maltotriose, maltulose, melezitose, isomaltose, palatinose, and isomalto-oligosaccharides [12]. Regarding *Opp,* Agaphonov et al. showed that disruption of the *Opp MAL1* (a homologue of *Op MAL1*) resulted in colonies not growing on maltose [49]. Two *MAL* clusters have been also detected in *Aspergillus oryzae*, with only one of them proven functional [50].

### 3.2. *MAL* Clusters Are Also Present in the Genomes of Other Non-Conventional Yeasts

In addition to *Op* and *Opp*, *MAL* clusters have been identified in some other non-conventional yeasts such as *S. stipitis* [21] and *Kluyveromyces lactis* [51,52] as well as in *Aspergillus* fungi [50]. In current study, *MAL* clusters of varied content, position, and number were detected in the genomes of *L. elongisporus*, *Torulaspora delbrueckii*, *M. guillermondii*, *C. fabianii*, *D. hansenii*, and *Lipomyces starkeyi* (Figure 1) but not in *B. adeninivorans* and *S. pombe*. *L. starkeyi* was most special with eight *MAL* clusters discovered. Notably, most of the *MAL* genes of *L. starkeyi* had introns (see Tables S1 and S2). In *S. stipitis*, introns were present only in putative *MAL*-activator genes *SUC1.1*, *SUC1.2*, and *SUC1.4*, no introns were reported for *MAL* genes of other studied yeasts. Subtelomeric positioning was verified for *MAL* loci of *D. hansenii* and *T. delbrueckii* (Figure 1), and for two *MAL* clusters of *S. stipitis* [21].

### 3.3. *MAL*-Activator Genes Are Often Genomically Clustered with *AG* and *AGT* Genes

*MAL*-activator is a positive transcriptional regulator of *MAL* genes containing an N-terminal Zn(2)-Cys(6) DNA-binding domain [53,54,55]. Many laboratory strains of *S. cerevisiae* (such as S288C and W303-1A) fail to grow on maltose because of a defective *MAL*-activator allele [23,56,57]. Until now, functionality of the *MAL*-activators of *S. cerevisiae* (MALx3 proteins) and *Candida albicans* (SUC1 protein) has been proven [53,54,55,58]. Figure 1 (pink arrows) depicts potential *MAL*-activator genes in *MAL* clusters of yeasts and *Aspergillus*. Notably, while most *MAL* clusters have a divergently positioned pair of *AG* and *AGT* genes, the position and transcription direction of *MAL*-activator genes varies between the clusters. The *MAL* loci of both *Op* and *Opp* contain two potential *MAL*-activator genes with functionality and roles being yet not known. Our search did not detect potential *MAL*-activator genes in *MAL* clusters of *L. elongisporus* and *T. delbrueckii*.

### 3.4. Phylograms of AGTs and AGs Encoded by the *MAL* Clusters Largely Agree with Phylograms of Yeast Species

Our dataset of yeast genomes encompasses Ascomycota species of varied evolutionary age. The Ascomycota phylum is comprised of three monophyletic subphyla [59]: the Saccharomycotina, the Pezizomycotina (comprising filamentous fungi such as *Aspergillus* and *Neurospora*), and the Taphrinomycotina (syn. Archaeascomycota, comprising for example *S. pombe*). *Schizosaccharomyces* is most distant from other Ascomycota—evolutionary distance between *Schizosaccharomyces* and *Saccharomyces* is about 1 billion years, which is ~25% of the age of the Earth. The Saccharomycotina subphylum contains four major clades: (i) the Saccharomycetaceae; (ii) the CTG yeasts; (iii) the methylotrophs; and (iv) the basal group to the Saccharomycotina. The basal group that includes *Lipomyces* and *Blastobotrys* that were studied in current work is very heterogeneous according to their genomic signatures and proteomes [60].

Phylogenesis of AGTs and AGs was assayed as described in the Materials and Methods section. Proteins encoded in *MAL* clusters (Figure 1) were all included except for YIC1 (see Figure 1), a 823 aa protein annotated as a GH31 family α-glucosidase/xylosidase. In the case of *B. adeninivorans* and *S. pombe* in which we did not detect the *MAL* clusters, either proteins which have been experimentally studied (Sut1 and Mal1 of *S. pombe*) or closest homologues of respective *Op* proteins were added to the dataset. As the Sut1 transporter of *S. pombe* has a very low identity to other AGTs (Table S4), it was excluded from the phylogram. The Sut1 [61] is a fungal homologue of plant sucrose transporters that also has a considerable similarity to sugar transporters of bacteria.

**Figure 2:**
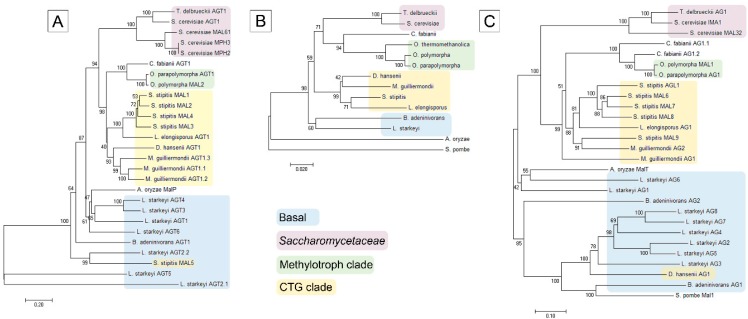
Phylogram of α-glucoside transporters (AGT) (**A**); α-glucosidases (AG) (**C**); and respective host species (**B**). See the Materials and Methods section for details. The scale bar in panels (**A**,**C**) indicates the number of substitutions per amino acid site, the scale bar in panel (**B**) indicates the number of base substitutions per site. The subgroups of Saccharomycotina according to [60] are designated by different background coloring.

In general, the phylogram of AGs coincided well with that of AGTs and D1/D2 sequences of ribosomal large subunit RNA of respective yeasts (Figure 2), suggesting that the *MAL* genes have evolved concomitantly with the yeast species. AGTs and AGs of *Op* and *Opp* clustered most closely with respective proteins of *C. fabianii.* AGTs and AGs of CTG yeasts (*S. stipitis, L. elongisporus* and *M. guillermondii*) [60,62] formed distinct clusters close to respective proteins of methylotrophs. AGTs and AGs of *S. pombe*, *B. adeninivorans* and *L. starkeyi* were revealed as early diverged representatives of these proteins. The phylogram of yeasts (Figure 2B) illustrates early branching of these three species. According to the literature, the family Lipomycetaceae is the earliest-branching lineage of Saccharomycotina, followed by a clade containing also *B. adeninivorans* [59].

In two cases, we detected clustering of AGs and AGTs that did not match the evolution of yeast species—the MAL5 permease of *S. stipitis* and AG1 of *D. hansenii* clustered within respective proteins of basal clade to Saccharomycotina.

### 3.5. Analysis of Yeast AGs for Signature Amino Acids: Prediction of Substrate Specificity

In most cases, the AGs encoded by *MAL* clusters (Figure 1) have not been studied for functionality and properties. However, substrate specificity of an AG can, at least to some extent, be predicted on the basis of amino acid residues residing close to the active site pocket [12,13,63,64]. Crystal structure is available for *S*. *cerevisiae* (*Sc*) isomaltase IMA1 and its catalytically inactive mutant. Respective structures in complex with maltose [64] or isomaltose and maltose [65], have revealed the amino acids Y158, V216, G217, S218, L219, M278, Q279, D307, and E411 bordering the substrate-binding pocket. These nine amino acids are variable between the maltases, isomaltases, and maltase-isomaltases, and have been used as a signature sequence for AGs [12,13,63,64]. A shared signature sequence for *Sc* IMA1 and IMA2 was YVGSLMQDE (see Table 2). Mutation of *Sc* IMA1 has shown that Val216 (shown in bold in the signature sequence) is of key importance for the ability for the enzyme to use isomaltose and isomaltose-like sugars [63,65]. All *Sc* isomaltases had a Val at respective positions (Table 2) and they hydrolyze isomaltose and isomaltose-like sugars (for example palatinose and α-MG), but not maltose [66]. If Val216 was mutated to a Thr (*Sc* maltases have a Thr at this position), the ability to hydrolyze maltose emerged in IMA1 and isomaltose hydrolysis concomitantly decreased [63]. Mutation of Gly217 and Ser219 in IMA1 to maltase-specific amino acids Ala and Gly, respectively, had additional effects on substrate specificity, increasing the maltose/isomaltose hydrolyzing ratio [63]. Substitution of Gln279 in IMA1 with Ala also shifted substrate specificity—hydrolysis of isomaltose was strongly reduced and a low maltose-hydrolyzing activity emerged [63,65]. Maltase-isomaltases of *Op* (MAL1) and *L. elongisporus* (we designated it *Le* AG1) use both maltose- and isomaltose-like substrates [12,13]. Similarly to *Sc* maltase, these proteins had Thr, Ala, and Gly at positions 216–217–218 (*Sc* IMA1 numbering) respectively (Table 2). Thus, these amino acids are assumed to be required for the hydrolysis of maltose and maltose-like substrates by both maltases and maltase-isomaltases. Mutation of Thr200 (equivalent of Val216 of IMA1) in *Op* maltase-isomaltase to a Val strongly reduces hydrolysis of maltose-like substrates, making the enzyme more similar to isomaltases [12,13].

For prediction of function (substrate specificity) of AGs of non-conventional yeasts, the protein sequences were aligned and signature amino acids were extracted from the alignment. AGs of *Sc*, *Aspergillus*, *Fusarium* and *Bacillus* AGs were used for comparison (Table 2).

The AG of *C. fabianii* (*Cf* AG1.2), the closest neighbor of *Op* MAL1 in the phylogenesis tree (Figure 2C), has a signature HTAGLVGDN differing from that of *Op* MAL1 only in the first position, suggesting that AG1.2 of *C. fabianii* is a maltase-isomaltase. *Cf* AG1.1 was predicted by us as an isomaltase—its signature sequence has six matches with that of isomaltase IMA5 of *Sc*. We also suggested that MAL6, MAL7, MAL8, MAL9, and AGL1 of *S. stipitis* are all maltase-isomaltases. Their signature amino acids had the highest number of matches with respective positions of experimentally studied maltase-isomaltases of *O. polymorpha* and *L. elongisporus* [12,13]. According to our prediction, the AG2 of *M. guillermondii* is a maltase-isomaltase and AG1 is an isomaltase. Signature sequences of AGs of *S. pombe*, *L. starkeyi*, and *B. adeninivorans* significantly differed from those described above. These signature sequences were grouped according to three characteristic motifs. A T_216_V_217_ motif (IMA1 numbering) shown in red letters, was detected in *Ls* AG1, *Ls* AG6, and *Ba* AG2. The MalT protein of *A. oryzae* encoded in the *MAL* cluster (Figure 1) had a T_216_V_217_ motif in the signature sequence. According to [50] the MalT protein had PNPG-hydrolyzing activity, was induced at maltose growth, and was defined by the authors as a maltase. The *Ba* AG2 is also a maltase (our unpublished data). Therefore we suggested that AGs with a T_216_V_217_ motif in the signature sequence are maltases. An A_216_I_217_ motif (Table 2, in green letters) was detected in signature sequence of two experimentally characterized maltases—Mal1 of *S. pombe* [67] and α-1,4-glucosidase (maltase; BAA12704.1) of *B. stearothermophilus*. If Ala200 (equivalent to Val216 of IMA1) of *B. stearothermophilus* maltase was mutated to Val, the ability to hydrolyze maltose was lost [68]. Due to the presence of a A_216_I_217_ motif in the signature sequence, the *Ls* AG2, AG4, and AG5 were predicted as maltases. A V_216_I_217_ motif (shown in blue letters) is present in three experimentally studied AGs presented in Table 2: oligo-1,6-glucosidase (isomaltase) of *B. thermoglucosidasius* [68] and two isomaltases of filamentous fungi—AgdC of *Aspergillus niger* and Foagl1 of *F. oxysporum* [69]. If Val200 (corresponds to Val216 of IMA1) in *B. thermoglucosidasius* isomaltase was mutated to Ala, the ability to hydrolyze maltose emerged in the enzyme [68]. We predict that AG3, AG7, and AG8 of *L. starkeyi*, AG1 of *D. hansenii* and AG1 of *B. adeninivorans* are all isomaltases.

### 3.6. Substrate Specificity Evaluation of *Scheffersomyces stipitis* AGs: Verifying the Prediction

#### 3.6.1. *S. stipitis* Assimilates Both Maltose-Like and Isomaltose-Like Sugars

Several AGs from non-conventional yeasts, including five AGs of *S. stipitis*, were predicted by us as maltase-isomaltases (Table 2) meaning that they should enable the yeast to assimilate a wide range of α-glucosidic sugars. According to the CBS data, *S. stipitis* CBS 6054 assimilates glucose, galactose, xylose, sucrose, maltose, trehalose, melezitose, α-MG and few other sugars. However, the ability of *S. stipitis* to grow on many α-glucosidic sugars such as turanose, maltulose, maltotriose, isomaltose, and palatinose has not been previously studied. We assayed the growth of *S. stipitis* on these sugars using glucose, maltose, sucrose, xylose, and α-MG as reference substrates. All these sugars supported aerobic growth of *S. stipitis* showing that it assimilates not only sugars that are cleaved by maltases (maltose, maltotriose, turanose, maltulose), but also those cleaved by isomaltases (isomaltose, palatinose, α-MG) [12,13]. Therefore, this yeast must have enzymatic capacity to hydrolyze both maltose- and isomaltose-like sugars.

#### 3.6.2. Cell Extracts of *S. stipitis* Hydrolyzed both Maltose-Like and Isomaltose-Like Sugars

**Figure 3:**
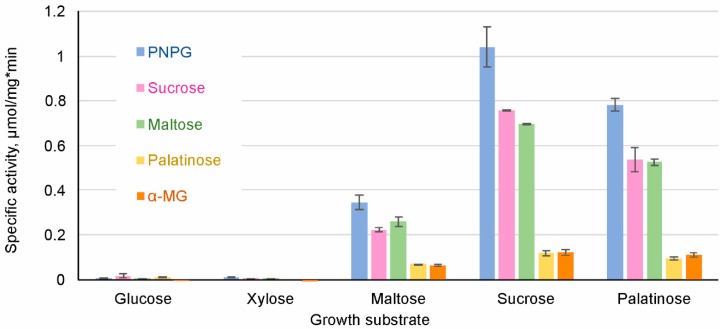
Specific activity of hydrolysis of α-glucosidic substrates in cell extracts of *S. stipitis* grown on different sugars. α-MG, α- methylglucoside; PNPG, *p*-nitrophenyl-α-d-glucopyranoside.

*S. stipitis* was grown on glucose, sucrose, maltose, palatinose and xylose as a single carbon source, all supplemented at 1%. Cell extracts were assayed for the hydrolysis of 1 mM PNPG and 100 mM maltose, sucrose, palatinose and α-MG (Figure 3).

The extracts of cells grown on either glucose or xylose had only negligible ability to hydrolyze PNPG, sucrose, maltose, palatinose and α-MG (Figure 3). High AG activity was observed in the cells grown on maltose, palatinose, or sucrose. Sucrose was the most potent inducer of AG activity, followed by palatinose (an isomaltose-like sugar). In some yeasts such as *S. cerevisiae*, sucrose can be hydrolyzed also by invertase (β-fructofuranosidase). AGs and invertases can be differentiated by the inability of AGs to hydrolyze raffinose [70]. As we did not detect raffinose-hydrolyzing activity in cell extracts of *S. stipitis*, sucrose hydrolysis measured in *S. stipitis* extracts was due to AG activity.

Repression of AGs by glucose has been described in several yeasts such as *S. cerevisiae*, *O. polymorpha*, and *C. albicans* [5,71,72]. Our data showed that xylose also repressed AGs expression. In accordance with our data, Jeffries et al. [25] detected the expression of only one *AG* gene (*MAL8*) in xylose-grown *S. stipitis*, and the level of expression was very low. Expression of *MAL8* was not detected in glucose-grown *S. stipitis* [25].

#### 3.6.3. MAL7, MAL8, and MAL9 of *S. stipitis* Proven to be Maltase-Isomaltases

**Figure 4:**
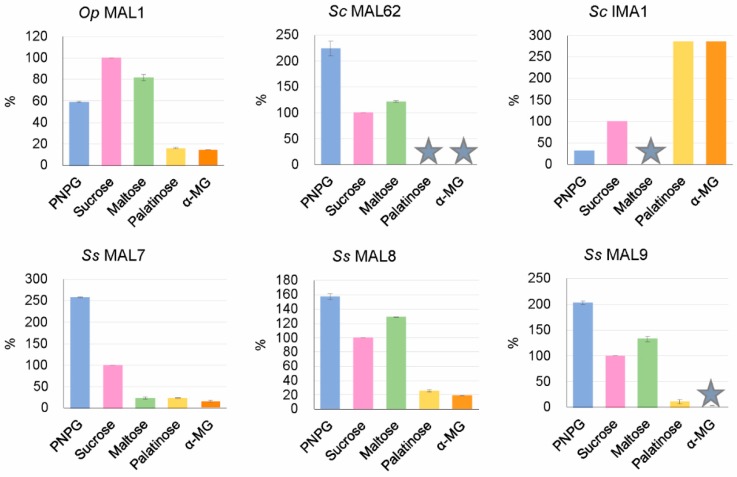
Substrate specificity of *S. stipitis* MAL7, MAL8, and MAL9 (lower panels) with *O. polymorpha* MAL1 (maltase-isomaltase), *S. cerevisiae* MAL62 (maltase), and IMA1 (isomaltase) (upper panels) used as references. Specific activity (µmol/mg·min) of each enzyme preparation with 100 mM sucrose was taken for 100%. Data on *Sc* IMA1 were calculated from Table 2 of [66], data on *Op* MAL1 were taken from Table 1 of [12]. The asterisk shows nondetectable or only negligible activity with corresponding substrates. Standard deviation values of the mean are given on the bars. See [66] for standard deviation data of *Sc* IMA1.

As mentioned above, *S. stipitis* AGs were predicted by us in silico as maltase-isomaltases (Table 2). To verify this prediction, we chose three of them (MAL7, MAL8, and MAL9) for heterologous expression in *E. coli* to study their substrate specificity. Extracts of recombinant *E. coli* were used as crude preparations to assay enzymatic hydrolysis of PNPG, maltose, sucrose, palatinose, and α-MG as described in Materials and Methods. The extract of *E. coli* transformant carrying the maltase gene *MAL62* of *S. cerevisiae* was used as a reference for maltase activity. In agreement with our earlier data [6], *E. coli* revealed no background AG activity. Results of substrate specificity assay are presented in Figure 4. Data regarding *Op* MAL1 (a maltase-isomaltase) and *Sc* IMA1 (an isomaltase) were taken from the literature [12,66].

Substrate specificity assay of *S. stipitis* MAL7, MAL8, and MAL9 (Figure 4) confirmed our prediction—all these enzymes hydrolyzed not only maltose-like substrates (maltose, sucrose) but also isomaltose-like substrates (palatinose, α-MG). *S. cerevisiae* MAL62 used as a reference, behaved as a typical maltase using only maltose-like substrates.

## 4. Discussion

### 4.1. The Natural Habitat of Non-Conventional Yeasts Possessing *MAL* Genes Contains α-Glucosidic Sugars

Yeasts typically prefer sugars to other carbon sources and therefore their natural habitats are sugar-rich. Plant sap and berries are rich in sucrose, whereas degradation of plant starch yields a variety of α-glucosidic oligosaccharides such as maltose, maltotriose, and isomaltose [12]. *Ogataea* species that use α-glucosidic sugars as well as methanol, have been isolated from spoiled plant material, plant leaves, soil, and insect gut. Living plant leaves emit methanol [73], raising from turnover of cell-wall pectin, and methanol is also released in soil due to the degradation of plant pectin and lignin [12]. *C. fabianii* has been isolated from alcoholic beverages, sugar cane (very rich in sucrose) and clinical material. The isolate YJS4271 analyzed in this work, was isolated from olives in Spain [33]. *M. guillermondii* is a flavinogenic yeast that grows on n-alkanes and pentoses, but also on α-glucosidic sugars [35,74]. Intriguingly, riboflavin metabolism and α-glucoside utilization are somehow linked in *M. guillermondii*. So, riboflavin permease was synthesized only in media containing α-glucosides, including sucrose, maltose, α-MG, and melezitose, whereas cells grown on other substrates (glucose, fructose) did not take up riboflavin [74]. *S. stipitis* can be isolated from the gut of passalid beetles that inhabit and degrade white-rotten hardwood [21]. According to our best knowledge, the current study is the first one on the metabolism of α-glucosidic sugars in *S. stipitis. D. hansenii* is a halo- and cryotolerant marine yeast found in cheese, dairy and brine [75]. A lipid-accumulating yeast *L. starkeyi* CBS 1807, from which we detected eight genomic *MAL* clusters (Figure 1), was isolated from soil, which is considered a primary habitat of this yeast [76]. Bacterial and fungal soil residents assumingly support *L. starkeyi* with α-glucosidic oligosaccharides arising from starch-rich plant residues. *B. adeninivorans* strains have been isolated from soil and wood hydrolysates. This yeast has great biotechnological potential because of metabolism of rare substrates such as *n*-butanol and plant phenolics. Metabolism of α-glucosidic oligosaccharides has not been assayed in *B. adeninivorans. S. pombe* has been isolated from fermented beverages, fruits, kombucha, and molasses used to produce rum and tequila [77]. Our current study shows that all above-mentioned yeasts have genes for transport and hydrolysis of α-glucosidic sugars. Mostly, these genes were found as constituents of genomic *MAL* clusters.

### 4.2. A Bi-Directional Promoter between the *AGT* and *AG* Genes Contributes to Balance the Transport and Further Metabolism of Disaccharides

In most *MAL* clusters of yeasts (Figure 1) as well as of numerous *Aspergillus* species [50], the genes for AGT and AG occur divergently positioned, sharing a bi-directional promoter. This genomic setup has been preserved in evolution as it enables tightly coordinated expression of both genes. Co-induction of these genes from a shared promoter by maltose and co-repression by glucose has been reported for *S. cerevisiae*, *O. polymorpha* and *A. oryzae* [10,50,72]. Induction of the promoter by maltose was shown to be stronger in AG direction in *Op*, whereas the opposite was true for *Sc* [72,78]. The AGT activity has been shown first required to provide intracellular maltose for the induction of *MAL* genes [79,80]. As *S. cerevisiae* strains usually have several *MAL* loci, they most likely have sufficiently high basal AGT activity to ensure the induction. Our data on *Op* indicated that monosaccharides (glucose and fructose) produced at intracellular hydrolysis of disaccharides repress the *MAL1* promoter and their phosphorylation is obligatory for the repression. At the same time, temporary accumulation of unphosphorylated hydrolysis products was shown to activate the promoter [11]. The growth of *Op* on disaccharides is assumingly complicated [11], because hydrolysis products of disaccharides that promote initial derepression of *MAL* genes will later cause repression when their phosphorylated species accumulate. Therefore, expression of AGT and AG proteins has to be finely adjusted with further glycolytic flux to provide an appropriate expression level of *MAL* genes. In good accordance with this, it was shown that *S. cerevisiae* cells (i) lyse if the transport and intracellular hydrolysis of maltose are not balanced [81]; and (ii) if grown at maltose limitation and then exposed to excess maltose, get rid of some intracellular glucose by efflux via glucose transporters [82].

### 4.3. How have the *MAL* Clusters Emerged and Evolved?

Among early diverged yeasts *S. pombe*, *B. adeninivorans* and *L. starkeyi*, *MAL* clusters were found only in *L. starkeyi*. *MAL* clusters have also been described in *Aspergillus* species [50] (belonging to Pezizomycotina, a subfamily of Ascomycota). We hypothesize that initially, a two-gene cluster comprised of divergently positioned *AG* and *AGT* genes was formed, and only after that was a *MAL*-activator gene added. As emphasized in [21], a divergent orientation (as in the case of *AG* and *AGT* genes) is typical for the genes that assumingly have evolved in longest association with one another. It is possible that the *MAL* clusters formed already before the separation of Pezizomycotina and Ascomycotina. Blastanalysis of AGs of *L. starkeyi MAL* clusters indicated that they were most similar to respective (putative) enzymes of Pezizomycotina species—*Aspergillus*, *Fusarium*, *Penicillium*, and others. Interestingly, the *MAL* cluster genes of *L. starkeyi* that belong to the most basal lineage of *Saccharomycotina*, were highly intronated. The genome of *L. starkey* is extremely rich in introns, with approximately three introns per protein-encoding gene [60]. Introns were also detected in *AG* genes of filamentous fungi *A. niger* and *F. oxysporum* (Table S1). According to [83,84], introns—a specific genetic feature of eukaryotes—existed at a high density in ancestral fungi and already in the last common ancestor of all extant eukaryotes. In many lineages of modern fungi (for example *Saccharomyces* and methylotrophs), massive loss of introns has been reported [60].

The bacterial origin of at least some *MAL* cluster genes can also be considered. According to Gabriško [85], although with low bootstrap values, fungal AGs of the glycoside hydrolase (GH) family 13 (GH13) always root deeply in the prokaryotic group, hinting that they may have bacterial ancestry. He also considers a possibility of (ancient) horizontal transfer of *AG* genes from bacteria. As bacteria do not have introns in protein-encoding genes, gain of introns (ancestral fungi are intron-rich) is expected. So, Da Lage et al. [84] showed that if an α-amylase gene from an actinobacterium was horizontally transferred to already intron-rich ancestor of Agaricomycotina fungi, introns were inserted soon after the transfer to adjust with requirements of the splicing machinery of the host.

Our current study (Table 2) clearly shows that AGs of early diverged yeasts *Lipomyces*, *Blastobotrys*, and *Schizosaccharomyces* have more similarity to AGs of filamentous fungi (Pezizomycotina) and bacteria (*Bacillus*) than to AGs of more recently diverged yeast lineages. Intriguingly, the *MAL1* gene of *O. polymorpha* also has some features of a bacterial gene—its promoter region is perfectly recognized in bacteria as possesses two pairs of sigma 70-like sequences [8]. Due to that, the *MAL1* promoter was successfully applied for overexpression of foreign proteins in *E. coli* [86].

### 4.4. Evolution of AGs: Repeated Changes in Substrate Specificity

We suppose that substrate specificity of AGs has been repeatedly altered during the evolution. According to the literature data (see Introduction) and our current study, specialized AGs—maltases and isomaltases—are found not only in ‘modern’ yeasts (*S. cerevisiae*), but also in early diverged Saccharomycotina species *L. starkeyi* and *B. adeninivorans*. In *O. polymorpha*, *L. elongisporus* [12,13], and *S. stipitis* (Figure 4) having intermediate position in Ascomycota phylogenesis (Figure 2B), promiscuous AGs have been described. Gabriško [85] suggested that (i) the common ancestor of the Ascomycota phylum had two specialized *AG* genes; (ii) in the subphylum Saccharomycotina, one of these genes (coding for isomaltase) was lost and the other one (coding for maltase) was retained and further duplicated in the lineage; (iii) in Pezizomycotina (filamentous fungi) evolution, both maltase and isomaltase genes were retained and one of them (coding for isomaltase) was duplicated. Considering that, filamentous fungi are expected to have more isomaltases than maltases. According to Gabriško [85], in distinct lineages of the Saccharomycotina, isomaltases evolved repeatedly from maltases, meaning that maltase-isomaltases found in *O. polymorpha*, *L. elongisporus*, and *S. stipitis* may represent ‘half-way’ variants of AGs evolution. As shown in [13] subsequent divergent evolution of promiscuous AGs can give rise to specific AGs—maltases and isomaltases. Most likely, the spectrum and amount of α-glucosidic oligosaccharides in yeasts’ habitat act as main driving force in substrate specificity evolution of AGs.

## 5. Concluding Remarks

Genome mining of non-conventional yeasts revealed *MAL* clusters that encode proteins most of which have not been characterized. AGs encoded by *MAL* clusters and elsewhere in the genome are perfect objects to study protein evolution, but they may also have biotechnological value. We detected eight *MAL* clusters in a lipid-accumulating yeast *L. starkeyi*, all encoding a potential AG protein. Since now, only one paper on an AG of *L. starkeyi* [87] has been published. It would be highly interesting to purify and biochemically characterize AGs of early diverged yeasts (*L. starkeyi* and *B. adeninivorans*) for their comparison with AGs of filamentous fungi and bacteria. *S. stipitis* has mostly been studied from the aspect of metabolism of lignocellulosic sugars and production of biofuel [25]. We showed that aside from xylose, glucose, mannose, galactose, cellobiose, and xylose oligomers derived from lignocellulosic material, *S. stipitis* assimilates numerous α-glucosidic sugars. This knowledge can be used for biotechnological purposes when selecting substrates for cultivation of *S. stipitis*.

## Figures and Tables

**Table 1:** Yeast strains and genomes analyzed in the current study.

Yeast	Culture Collection Numbers	Genome Accession from	Reference
*Ogataea polymorpha* leu1.1	NCYC 495; ATCC MYA-335; CBS 1976, NRRL Y-1789	MycoCosm	[30]
*Ogataea parapolymorpha* DL-1	ATCC 26012; CBS 12304; NRRL Y-7560	MycoCosm	[26]
*Lipomyces starkeyi*	NRRL Y-11557; ATCC 58680; CBS 1807	MycoCosm	[30]
*Debaryomyces hansenii*	CBS 767; ATCC 36239	MycoCosm	[33,34]
*Meyerozyma (Pichia) guillermondii*	CBS 566; ATCC 6260	MycoCosm	[32,35]
*Scheffersomyces* (*Pichia*) *stipitis*	CBS 6054	MycoCosm	[25]
*Lodderomyces elongisporus*	NRRL YB-4239; CBS 2605; ATCC 11503	MycoCosm	[32]
*Blastobotrys* (*Arxula*) *adeninivorans* LS3	CBS 8244	MycoCosm	[20,33]
*Schizosaccharomyces pombe*	ATCC 24843, CBS 10395	MycoCosm	[36]
*Cyberlindnera fabianii*	YJS4271	European Nucleotide Archive (ENA)	[37]
*Torulaspora delbrueckii*	CBS 1146	MycoCosm	[38]
*Saccharomyces cerevisiae* S288C	CBS 8803; ATCC 204508	MycoCosm	[39]

**Table 2:** Signature amino acids of α-glucosidases (AGs) and prediction of substrate specificity. Background coloring is as follows: maltases (yellow), isomaltases (blue), maltase-isomaltases (green), enzymes not studied experimentally before this study (white). The key position of *Sc* IMA1 (Val216) is shown in bold. Characteristic motifs T_216_V_217_, A_216_I_217_, and V_216_I_217_ of AGs are shown in red, green, and blue letters, respectively. *Bs*, *Bacillus stearothermophilus*; *Bt*, *Bacillus thermoglucosidasius*; *Ao*, *Aspergillus oryzae*; *Fo*, *Fusarium oxysporum*; *An*, *Aspergillus niger*. See Table S1 for acronyms of AGs.

α-Glucosidase	Signature Amino Acids (Numbering as in *Sc* IMA1)									Function (Prediction)
	158	216	217	218	219	278	279	307	411	
*Op* MAL1/*Opp* AG1	F	**T**	A	G	L	V	G	D	N	maltase-isomaltase
*Le* AG1	H	**T**	A	G	M	V	G	D	N	maltase-isomaltase
ancMALS	F	**T**	A	G	L	V	G	D	E	maltase-isomaltase
*Sc* MAL12/*Sc* MAL32/Sc MAL62	F	**T**	A	G	L	V	A	E	D	maltase
*Sc* IMA1/*Sc* IMA2	Y	**V**	G	S	L	M	Q	D	E	isomaltase
*Sc* IMA3/4	Y	**V**	G	S	L	M	R	D	E	isomaltase
*Sc* IMA5	F	**V**	G	S	M	V	G	S	E	isomaltase
*Td* AG1	Y	**V**	G	S	L	M	Q	D	E	isomaltase
*Cf* AG1.2	H	**T**	A	G	L	V	G	D	N	maltase-isomaltase
*Cf* AG1.1	M	**V**	C	S	L	V	G	S	Q	isomaltase
*Ss* MAL6	Y	**T**	A	G	L	V	G	N	N	maltase-isomaltase
*Ss* MAL7	F	**T**	A	G	L	V	G	T	N	maltase-isomaltase
*Ss* MAL8	Y	**T**	A	G	L	V	G	E	N	maltase-isomaltase
*Ss* MAL9	Y	**T**	A	G	M	V	G	E	N	maltase-isomaltase
*Ss* AGL1	Y	**T**	A	G	L	V	G	W	N	maltase-isomaltase
*Mg* AG2	Y	**T**	A	G	M	V	G	D	N	maltase-isomaltase
*Mg* AG1	C	**V**	A	A	L	V	G	E	E	isomaltase
*Ls* AG1	Y	**T**	V	N	K	L	S	H	E	maltase
*Ls* AG6	N	**T**	V	N	R	L	P	G	R	maltase
*Ba* AG2	Y	**T**	V	Q	I	G	S	R	N	maltase
*Ao* MalT	I	**T**	V	N	M	L	P	D	D	maltase
*Ls* AG2	L	**A**	I	N	F	M	A	D	E	maltase
*Ls* AG4	H	**A**	I	N	F	M	G	T	E	maltase
*Ls* AG5	A	**A**	I	N	F	M	A	D	E	maltase
*Sp* Mal1	Y	**A**	I	N	M	M	P	D	E	maltase
*Bs* α-1,4-glucosidase	I	**A**	I	S	H	A	N	G	A	maltase
*Ls* AG3	C	**V**	I	N	F	M	P	D	E	isomaltase
*Ls* AG7	E	**V**	I	N	Y	M	G	Q	E	isomaltase
*Ls* AG8	-	**V**	I	N	F	M	P	D	E	isomaltase
*Dh* AG1	A	**V**	I	N	F	M	P	D	E	isomaltase
*Ba* AG1	Y	**V**	I	N	L	M	P	Q	E	isomaltase
*Bt* oligo-1,6-glucosidase	V	**V**	I	N	M	T	P	D	E	isomaltase
*An* AgdC	F	**V**	I	N	F	M	P	D	D	isomaltase
*Fo* Foagl1	F	**V**	I	N	F	M	P	D	D	isomaltase

## Supplementary Materials

The following are available online at http://www.mdpi.com/2073-4425/9/7/354/s1, Table S1: α-glucosidases (AGs) used in current study, Table S2: α-glucoside transporters (AGTs) used in current study Table S3: Identity matrix of α-glucosidases (AGs) of yeasts and bacilli, Table S4: Identity matrix of yeast α-glucoside transporters (AGTs), Table S5. Primers for amplification of *Scheffersomyces stipitis* α-glucosidase genes of the *MAL* loci, Table S6. Primers for cloning the *Scheffersomyces stipitis* α-glucosidase genes to pURI3-Cter vector, Table S7. Synteny between the *Ogataea polymorpha* NCYC 495 and *Ogataea parapolymorpha* DL-1 genomes according to MycoCosm data. Forward synteny between the chromosomes is shown on white background, reverse syntheny on grey background, Table S8. Hypothetical permease and α-glucosidase genes outside the *MAL* locus in *Ogataea polymorpha* and *Ogataea parapolymorpha* with their genomic neighbors indicated. Transcription direction is indicated by arrow.
