# Supplementary material for: Genome Mining of Non-Conventional Yeasts: Search and Analysis of MAL Clusters and Proteins
Source: Genes (Basel). 2018 Jul 16;9(7):354. doi: 10.3390/genes9070354 (PMC6070925; doi:10.3390/genes9070354)
Supplement: Supplementary file 1 [file genes-09-00354-s001.pdf]

# Supporting information

## Genome mining of non-conventional yeasts: search and analysis of *MAL* clusters and proteins

Katrin Viigand[1], Kristina Põšnograjeva[1], Triinu Visnapuu, Tiina Alamäe*

Department of Genetics, Institute of Molecular and Cell Biology, University of Tartu, Riia 23, 51010 Tartu, Estonia

[1] Equal contributionThese authors contributed equally to this work

[*] Corresponding author: T. Alamäe, Department of Genetics, Institute of Molecular and Cell Biology, University of Tartu, Riia 23, 51010 Tartu, Estonia

Tel.: +372 7375013

E-mail: tiina@alamae.eu

**Table S1.** α-glucosidases (AGs) used in current study

| Yeast/Bacterium | Acronym for α-glucosidase | Mycocosm ID | GenBank ID | Protein length (aa) | No of introns |
|---|---|---|---|---|---|
| ***Blastobotrys adeninivorans*** | AG1 | rna_ARAD1C14212g | Not available | 567 | 0 |
| | AG2 | rna_ARAD1D20130g | Not available | 581 | 0 |
| ***Lipomyces starkeyi*** | AG1 | gm1.3262_g | ODQ72902 | 582 | 2 |
| | AG2 | fgenesh1_kg.8_#_10_#_Locus10580v1rpkm1.26 | ODQ71836.1 | 621 | 4 |
| | AG3 | fgenesh1_kg.21_#_1_#_Locus3996v1rpkm38.55 | ODQ68842 | 582 | 6 |
| | AG4 | CE77125_4419 | ODQ69382.1 | 578 | 4 |
| | AG5 | fgenesh1_kg.10_#_105_#_Locus4759v1rpkm25.91 | ODQ71069.1 | 641 | 4 |
| | AG6 | fgenesh1_kg.6_#_581_#_Locus4531v6rpkm0.18_PRE | ODQ73295.1 | 574 | 0 |
| | AG7 | estExt_Genemark1.C_170108 | ODQ69164.1 | 629 | 1 |
| | AG8 | gm1.6616_g | ODQ69958.1 | 555 | 1 |
| ***Cyberlindnera fabianii*** | AG1.1 | Not available | CDR39670.1 | 563 | 0 |
| | AG1.2 | Not available | CDR39674.1 | 563 | 0 |
| ***Meyerozyma guilliermondii*** | AG1 | PGUG_01643T0 | EDK37545.2 | 567 | 0 |
| | AG2 | PGUG_05731T0 | XP_001481968.1 | 568 | 0 |
| ***Torulaspora delbrueckii*** | AG1 | TDEL_0D06530 | XP_003681448.1 | 604 | 0 |
| ***Saccharomyces cerevisiae*** | MAL32 | YBR299W | NP_009858.3 | 584 | 0 |
| | IMA1 | YGR287C | NP_011803.3 | 589 | 0 |
| ***Ogataea polymorpha*** | MAL1 | fgenesh1_kg.1_#_333_#_isotig01325 | XP_018213389.1 | 564 | 0 |
| ***Ogataea parapolymorpha*** | AG1 | HPODL_02607T0 | XP_013932394 | 564 | 0 |
| ***Scheffersomyces stipitis*** | AGL1 | e_gww1.6.1.354.1 | XP_001385341.1 | 572 | 0 |
| | MAL6 | e_gwh1.2.1.484.1 | XP_001382912.2 | 572 | 0 |
| | MAL7 | e_gww1.5.1.399.1 | XP_001384657.2 | 572 | 0 |
| | MAL8 | estExt_genewise1_worm.C_chr_6.10336 | XP_001385796.1 | 572 | 0 |
| | MAL9 | fgenesh1_pg.C_chr_1.2000465 | XP_001387228.2 | 573 | 0 |
| ***Lodderomyces elongisporus*** | AG1 | LELG_01359T0 | XP_001526531.1 | 585 | 0 |
| ***Debaryomyces hansenii*** | AG1 | Not available | XP_459350.2 | 578 | 0 |
| ***Schizosaccharomyces pombe*** | Mal1 | mal1 | NP_595063.1 | 579 | 0 |
| ***Bacillus stearothermophilus*** | α-1,4-glucosidase | Not applicable | BAA12704.1 | 555 | 0 |
| ***Bacillus thermoglucosidasius*** | oligo-1,6-glucosidase | Not applicable | P29094.1 | 562 | 0 |
| ***Aspergillus oryzae*** | MalT | AO090038000234_mRNA | XP_001825184.1 | 574 | 0 |
| ***Aspergillus niger*** | AgdC | An02g13240m.01 | XP_001400455.1 | 587 | 2 |
| ***Fusarium oxysporum*** | Foagl1 | FOXG_00152T1 | XP_018231823 | 571 | 4 |

**Table S2.** α-glucoside transporters (AGTs) used in current study

| Yeast | Acronym for α-glucoside transporter | Mycocosm ID | GenBank ID | Protein length (aa) | No of introns |
|---|---|---|---|---|---|
| ***Blastobotrys adeninivorans*** | AGT1 | rna_ARAD1D09658g | Not available | 534 | 0 |
| ***Lipomyces starkeyi*** | AGT1 | estExt_Genewise1.C_6_t10076 | ODQ72903 | 510 | 0 |
| | AGT2.1 | gm1.4217_g | ODQ71837.1 | 511 | 1 |
| | AGT2.2 | estExt_Genewise1.C_8_t10022 | ODQ71838.1 | 498 | 0 |
| | AGT3 | estExt_Genemark1.C_210003 | ODQ68844 | 533 | 1 |
| | AGT4 | fgenesh1_kg.16_#_243_#_Locus11337v1rpkm1.02 | ODQ69380.1 | 616 | 0 |
| | AGT5 | gm1.5097_g | ODQ71068.1 | 527 | 1 |
| | AGT6 | gm1.3625_g | ODQ73296.1 | 544 | 1 |
| ***Cyberlindnera fabianii*** | AGT1 | Not available | CDR39675.1 | 582 | 0 |
| ***Meyerozyma guilliermondii*** | AGT1.1 | PGUG_01640T0 | EDK37542.2 | 579 | 0 |
| | AGT1.2 | PGUG_01641T0 | EDK37543.2 | 585 | 0 |
| | AGT1.3 | PGUG_01642T0 | XP_001485971.1 | 574 | 0 |
| ***Torulaspora delbrueckii*** | AGT1 | TDEL_0D06520 | XP_003681447.1 | 614 | 0 |
| ***Saccharomyces cerevisiae*** | MAL61* | NA | P15685.1 | 614 | 0 |
| | AGT1 | YGR289C | NP_011805.3 | 616 | 0 |
| | MPH2 | YDL247W | P0CD99.1 | 609 | 0 |
| | MPH3 | YJR160C | P0CE00.1 | 602 | 0 |
| ***Ogataea polymorpha*** | MAL2 | fgenesh1_kg.1_#_334_#_isotig00883 | XP_018213391.1 | 522 | 0 |
| ***Ogataea parapolymorpha*** | AGT1 | HPODL_02606T0 | XP_013932393 | 582 | 0 |
| ***Scheffersomyces stipitis*** | MAL1 | fgenesh1_pg.C_chr_2.1000315 | XP_001382383.1 | 581 | 0 |
| | MAL2 | fgenesh1_pg.C_chr_5.1000002 | XP_001385023.1 | 585 | 0 |
| | MAL3 | e_gwh1.6.1.324.1 | XP_001385693.1 | 581 | 0 |
| | MAL4 | e_gwh1.6.1.358.1 | XP_001385456.1 | 583 | 0 |
| | MAL5 | fgenesh1_pg.C_chr_6.1000012 | XP_001385340.2 | 509 | 0 |
| ***Lodderomyces elongisporus*** | AGT1 | LELG_01361T0 | XP_001526533.1 | 584 | 0 |
| ***Debaryomyces hansenii*** | AGT1 | DEHA2E00550g | XP_459351.2 | 582 | 0 |
| ***Schizosaccharomyces pombe*** | Sut1 | sut1 | NP_001342860.1 | 553 | 1 |
| ***Aspergillus oryzae*** | MalP | AO090038000233_mRNA | XP_001825183.1 | 539 | 0 |

* Strain CB11

**Table S3. Identity matrix of α-glucosidases (AGs) of yeasts and bacilli**

| | *T. delbrueckii* AG1 | *S. cerevisiae* MAL32 | *S. cerevisiae* IMA1 | *C. fabianii* AG1.1 | *C. fabianii* AG1.2 | *O. parapolymorpha* AG1 | *O. polymorpha* MAL1 | *M. guilliermondii* AG1 | *M. guilliermondii* AG2 | *L. elongisporus* AG1 | *S. stipitis* AGL1 | *S. stipitis* MAL6 | *S. stipitis* MAL7 | *S. stipitis* MAL8 | *S. stipitis* MAL9 | *D. hansenii* AG1 | *L. starkeyi* AG1 | *L. starkeyi* AG2 | *L. starkeyi* AG3 | *L. starkeyi* AG4 | *L. starkeyi* AG5 | *L. starkeyi* AG6 | *L. starkeyi* AG7 | *L. starkeyi* AG8 | *B. adeninivorans* AG1 | *B. adeninivorans* AG2 | *S. pombe* Mal1 | *A. oryzae* MalT | *A. niger* AgdC | *F. oxysporum* Foagl1 | *Bs α-1,4-glucosidase* | *Bt oligo-1,6-glucosidase* |
|---|---|---|---|---|---|---|---|---|---|---|---|---|---|---|---|---|---|---|---|---|---|---|---|---|---|---|---|---|---|---|---|---|
| *T. delbrueckii* AG1 | 100 | 68 | 81 | 42 | 41 | 41 | 41 | 47 | 49 | 46 | 47 | 47 | 46 | 47 | 46 | 41 | 44 | 34 | 37 | 36 | 34 | 44 | 34 | 35 | 38 | 41 | 37 | 43 | 37 | 35 | 35 | 40 |
| *S. cerevisiae* MAL32 | 68 | 100 | 72 | 42 | 44 | 43 | 43 | 47 | 49 | 46 | 49 | 49 | 49 | 47 | 48 | 41 | 44 | 34 | 36 | 36 | 33 | 44 | 34 | 36 | 40 | 41 | 38 | 43 | 36 | 35 | 37 | 39 |
| *S. cerevisiae* IMA1 | 81 | 72 | 100 | 44 | 43 | 44 | 44 | 49 | 50 | 48 | 49 | 51 | 49 | 50 | 49 | 44 | 45 | 34 | 38 | 37 | 34 | 46 | 35 | 35 | 40 | 44 | 38 | 44 | 37 | 36 | 36 | 42 |
| *C. fabianii* AG1.1 | 42 | 42 | 44 | 100 | 77 | 76 | 75 | 56 | 57 | 54 | 56 | 57 | 58 | 56 | 57 | 41 | 46 | 38 | 39 | 38 | 37 | 44 | 36 | 38 | 41 | 44 | 40 | 47 | 39 | 39 | 40 | 42 |
| *C. fabianii* AG1.2 | 41 | 44 | 43 | 77 | 100 | 86 | 86 | 56 | 58 | 57 | 59 | 58 | 58 | 59 | 56 | 43 | 46 | 38 | 38 | 38 | 37 | 43 | 38 | 39 | 40 | 46 | 40 | 47 | 39 | 40 | 41 | 42 |
| *O. parapolymorpha* AG1 | 41 | 43 | 44 | 76 | 86 | 100 | 98 | 57 | 59 | 56 | 59 | 60 | 60 | 59 | 57 | 41 | 46 | 38 | 38 | 37 | 36 | 43 | 36 | 38 | 39 | 44 | 40 | 45 | 38 | 38 | 40 | 41 |
| *O. polymorpha* MAL1 | 41 | 43 | 44 | 75 | 86 | 98 | 100 | 57 | 59 | 56 | 58 | 59 | 59 | 59 | 56 | 41 | 45 | 38 | 38 | 38 | 37 | 43 | 37 | 37 | 38 | 44 | 40 | 45 | 38 | 38 | 40 | 42 |
| *M. guilliermondii* AG1 | 47 | 47 | 49 | 56 | 56 | 57 | 57 | 100 | 62 | 59 | 63 | 64 | 62 | 63 | 60 | 45 | 51 | 39 | 41 | 41 | 39 | 46 | 39 | 39 | 42 | 48 | 43 | 51 | 43 | 42 | 39 | 44 |
| *M. guilliermondii* AG2 | 49 | 49 | 50 | 57 | 58 | 59 | 59 | 62 | 100 | 66 | 65 | 67 | 66 | 66 | 71 | 45 | 53 | 40 | 42 | 43 | 40 | 49 | 41 | 42 | 43 | 49 | 45 | 51 | 43 | 43 | 39 | 45 |
| *L. elongisporus* AG1 | 46 | 46 | 48 | 54 | 57 | 56 | 56 | 59 | 66 | 100 | 65 | 69 | 68 | 68 | 63 | 43 | 49 | 38 | 38 | 39 | 37 | 47 | 37 | 38 | 39 | 47 | 42 | 50 | 40 | 42 | 37 | 42 |
| *S. stipitis* AGL1 | 47 | 49 | 49 | 56 | 59 | 59 | 58 | 63 | 65 | 65 | 100 | 79 | 76 | 77 | 66 | 42 | 51 | 37 | 40 | 40 | 36 | 46 | 37 | 39 | 40 | 45 | 42 | 52 | 40 | 42 | 41 | 43 |
| *S. stipitis* MAL6 | 47 | 49 | 51 | 57 | 58 | 60 | 59 | 64 | 67 | 69 | 79 | 100 | 85 | 83 | 68 | 43 | 52 | 39 | 40 | 40 | 39 | 47 | 38 | 39 | 42 | 44 | 44 | 53 | 41 | 43 | 39 | 44 |
| *S. stipitis* MAL7 | 46 | 49 | 49 | 58 | 58 | 60 | 59 | 62 | 66 | 68 | 76 | 85 | 100 | 79 | 68 | 43 | 51 | 38 | 40 | 41 | 37 | 47 | 38 | 39 | 40 | 45 | 42 | 53 | 41 | 43 | 40 | 44 |
| *S. stipitis* MAL8 | 47 | 47 | 50 | 56 | 59 | 59 | 59 | 63 | 66 | 68 | 77 | 83 | 79 | 100 | 66 | 44 | 50 | 38 | 40 | 41 | 39 | 47 | 38 | 39 | 41 | 45 | 44 | 52 | 42 | 43 | 39 | 43 |
| *S. stipitis* MAL9 | 46 | 48 | 49 | 57 | 56 | 57 | 56 | 60 | 71 | 63 | 66 | 68 | 68 | 66 | 100 | 43 | 50 | 38 | 42 | 43 | 38 | 48 | 38 | 40 | 41 | 48 | 43 | 51 | 41 | 42 | 40 | 43 |
| *D. hansenii* AG1 | 41 | 41 | 44 | 41 | 43 | 41 | 41 | 45 | 45 | 43 | 42 | 43 | 43 | 44 | 43 | 100 | 45 | 51 | 56 | 59 | 52 | 43 | 52 | 58 | 49 | 45 | 51 | 43 | 54 | 53 | 41 | 46 |
| *L. starkeyi* AG1 | 44 | 44 | 45 | 46 | 46 | 46 | 45 | 51 | 53 | 49 | 51 | 52 | 51 | 50 | 50 | 45 | 100 | 42 | 43 | 47 | 41 | 54 | 41 | 43 | 43 | 50 | 45 | 55 | 45 | 44 | 40 | 43 |
| *L. starkeyi* AG2 | 34 | 34 | 34 | 38 | 38 | 38 | 38 | 39 | 40 | 38 | 37 | 39 | 38 | 38 | 38 | 51 | 42 | 100 | 53 | 59 | 74 | 39 | 56 | 58 | 42 | 40 | 45 | 42 | 46 | 46 | 36 | 42 |
| *L. starkeyi* AG3 | 37 | 36 | 38 | 39 | 38 | 38 | 38 | 41 | 42 | 38 | 40 | 40 | 40 | 40 | 42 | 56 | 43 | 53 | 100 | 58 | 53 | 43 | 53 | 58 | 49 | 44 | 49 | 44 | 56 | 55 | 39 | 47 |
| *L. starkeyi* AG4 | 36 | 36 | 37 | 38 | 38 | 37 | 38 | 41 | 43 | 39 | 40 | 40 | 41 | 41 | 43 | 59 | 47 | 59 | 58 | 100 | 59 | 43 | 58 | 64 | 50 | 44 | 51 | 45 | 55 | 52 | 40 | 48 |
| *L. starkeyi* AG5 | 34 | 33 | 34 | 37 | 37 | 36 | 37 | 39 | 40 | 37 | 36 | 39 | 37 | 39 | 38 | 52 | 41 | 74 | 53 | 59 | 100 | 39 | 57 | 58 | 44 | 40 | 45 | 40 | 48 | 46 | 36 | 42 |
| *L. starkeyi* AG6 | 44 | 44 | 46 | 44 | 43 | 43 | 43 | 46 | 49 | 47 | 46 | 47 | 47 | 47 | 48 | 43 | 54 | 39 | 43 | 43 | 39 | 100 | 41 | 41 | 44 | 49 | 41 | 56 | 44 | 42 | 39 | 41 |
| *L. starkeyi* AG7 | 34 | 34 | 35 | 36 | 38 | 36 | 37 | 39 | 41 | 37 | 37 | 38 | 38 | 38 | 38 | 52 | 41 | 56 | 53 | 58 | 57 | 41 | 100 | 69 | 45 | 41 | 45 | 43 | 49 | 49 | 37 | 42 |
| *L. starkeyi* AG8 | 35 | 36 | 35 | 38 | 39 | 38 | 37 | 39 | 42 | 38 | 39 | 39 | 39 | 39 | 40 | 58 | 43 | 58 | 58 | 64 | 58 | 41 | 69 | 100 | 49 | 42 | 49 | 44 | 52 | 53 | 40 | 45 |
| *B. adeninivorans* AG1 | 38 | 40 | 40 | 41 | 40 | 39 | 38 | 42 | 43 | 39 | 40 | 42 | 40 | 41 | 41 | 49 | 43 | 42 | 49 | 50 | 44 | 44 | 45 | 49 | 100 | 46 | 57 | 44 | 49 | 47 | 39 | 44 |
| *B. adeninivorans* AG2 | 41 | 41 | 44 | 44 | 46 | 44 | 44 | 48 | 49 | 47 | 45 | 44 | 45 | 45 | 48 | 45 | 50 | 40 | 44 | 44 | 40 | 49 | 41 | 42 | 46 | 100 | 41 | 49 | 44 | 41 | 38 | 40 |
| *S. pombe* Mal1 | 37 | 38 | 38 | 40 | 40 | 40 | 40 | 43 | 45 | 42 | 42 | 44 | 42 | 44 | 43 | 51 | 45 | 45 | 49 | 51 | 45 | 41 | 45 | 49 | 57 | 41 | 100 | 42 | 50 | 49 | 39 | 42 |
| *A. oryzae* MalT | 43 | 43 | 44 | 47 | 47 | 45 | 45 | 51 | 51 | 50 | 52 | 53 | 53 | 52 | 51 | 43 | 55 | 42 | 44 | 45 | 40 | 56 | 43 | 44 | 44 | 49 | 42 | 100 | 44 | 44 | 41 | 42 |
| *A. niger* AgdC | 37 | 36 | 37 | 39 | 39 | 38 | 38 | 43 | 43 | 40 | 40 | 41 | 41 | 42 | 41 | 54 | 45 | 46 | 56 | 55 | 48 | 44 | 49 | 52 | 49 | 44 | 50 | 44 | 100 | 57 | 39 | 45 |
| *F. oxysporum* Foagl1 | 35 | 35 | 36 | 39 | 40 | 38 | 38 | 42 | 43 | 42 | 42 | 43 | 43 | 43 | 42 | 53 | 44 | 46 | 55 | 52 | 46 | 42 | 49 | 53 | 47 | 41 | 49 | 44 | 57 | 100 | 40 | 48 |
| *Bs* α-1,4-glucosidase | 35 | 37 | 36 | 40 | 41 | 40 | 40 | 39 | 39 | 37 | 41 | 39 | 40 | 39 | 40 | 41 | 40 | 36 | 39 | 40 | 36 | 39 | 37 | 40 | 39 | 38 | 39 | 41 | 39 | 40 | 100 | 56 |
| *Bt* oligo-1,6-glucosidase | 40 | 39 | 42 | 42 | 42 | 41 | 42 | 44 | 45 | 42 | 43 | 44 | 44 | 43 | 43 | 46 | 43 | 42 | 47 | 48 | 42 | 41 | 42 | 45 | 44 | 40 | 42 | 42 | 45 | 48 | 56 | 100 |

*Bs, Bacillus stearothermophilus; Bt, Bacillus thermoglucosidasius*

| **Table S4. Identity matrix of yeast α-glucoside transporters (AGTs)** | *T. delbrueckii* AGT1 | *S. cerevisiae* MAL61 | *S. cerevisiae* MPH2 | *S. cerevisiae* MPH3 | *S. cerevisiae* AGT1 | *C. fabianii* AGT1 | *O. parapolymorpha* AGT1 | *O. polymorpha* MAL2 | *M. guilliermondii* AGT1.1 | *M. guilliermondii* AGT1.2 | *M. guilliermondii* AGT1.3 | *L. elongisporus* AGT1 | *D. hansenii* AGT1 | *S. stipitis* MAL1 | *S. stipitis* MAL2 | *S. stipitis* MAL3 | *S. stipitis* MAL4 | *S. stipitis* MAL5 | *L. starkeyi* AGT1 | *L. starkeyi* AGT2.1 | *L. starkeyi* AGT2.2 | *L. starkeyi* AGT3 | *L. starkeyi* AGT4 | *L. starkeyi* AGT5 | *L. starkeyi* AGT6 | *A. oryzae* MalP | *B. adeninivorans* AGT1 | *S. pombe* Sut1 | *S. pombe* Ght3 |
|---|---|---|---|---|---|---|---|---|---|---|---|---|---|---|---|---|---|---|---|---|---|---|---|---|---|---|---|---|---|
| *T. delbrueckii* AGT1 | 100 | 53 | 52 | 52 | 67 | 36 | 36 | 35 | 39 | 37 | 37 | 36 | 36 | 36 | 36 | 37 | 35 | 26 | 32 | 14 | 29 | 33 | 34 | 18 | 34 | 33 | 32 | 14 | 9 |
| *S. cerevisiae* MAL61 | 53 | 100 | 73 | 74 | 54 | 36 | 38 | 37 | 42 | 40 | 39 | 38 | 37 | 38 | 39 | 38 | 38 | 27 | 36 | 17 | 29 | 32 | 35 | 19 | 36 | 36 | 32 | 16 | 8 |
| *S. cerevisiae* MPH2 | 52 | 73 | 100 | 96 | 51 | 36 | 39 | 37 | 40 | 40 | 38 | 39 | 38 | 37 | 38 | 38 | 36 | 27 | 37 | 16 | 29 | 33 | 36 | 19 | 36 | 37 | 31 | 16 | 7 |
| *S. cerevisiae* MPH3 | 52 | 74 | 96 | 100 | 52 | 36 | 39 | 38 | 41 | 40 | 38 | 39 | 38 | 37 | 38 | 37 | 36 | 28 | 37 | 16 | 30 | 33 | 36 | 19 | 37 | 37 | 31 | 16 | 7 |
| *S. cerevisiae* AGT1 | 67 | 54 | 51 | 52 | 100 | 38 | 37 | 37 | 39 | 39 | 35 | 37 | 37 | 37 | 38 | 37 | 37 | 28 | 35 | 15 | 28 | 33 | 36 | 20 | 34 | 35 | 31 | 16 | 8 |
| *C. fabianii* AGT1 | 36 | 36 | 36 | 36 | 38 | 100 | 73 | 67 | 57 | 55 | 54 | 51 | 49 | 55 | 51 | 52 | 54 | 30 | 41 | 17 | 31 | 41 | 42 | 20 | 41 | 41 | 38 | 16 | 9 |
| *O. parapolymorpha* AGT1 | 36 | 38 | 39 | 39 | 37 | 73 | 100 | 87 | 57 | 56 | 54 | 50 | 50 | 52 | 51 | 51 | 51 | 29 | 41 | 18 | 31 | 39 | 40 | 20 | 41 | 41 | 37 | 17 | 9 |
| *O. polymorpha* MAL2 | 35 | 37 | 37 | 38 | 37 | 67 | 87 | 100 | 54 | 53 | 53 | 48 | 47 | 50 | 49 | 49 | 49 | 33 | 45 | 19 | 35 | 40 | 41 | 20 | 41 | 42 | 38 | 18 | 9 |
| *M. guilliermondii* AGT1.1 | 39 | 42 | 40 | 41 | 39 | 57 | 57 | 54 | 100 | 79 | 69 | 55 | 56 | 58 | 56 | 54 | 55 | 30 | 43 | 18 | 34 | 40 | 43 | 22 | 42 | 42 | 40 | 17 | 10 |
| *M. guilliermondii* AGT1.2 | 37 | 40 | 40 | 40 | 39 | 55 | 56 | 53 | 79 | 100 | 68 | 56 | 55 | 55 | 55 | 52 | 56 | 31 | 41 | 18 | 34 | 39 | 42 | 22 | 42 | 41 | 38 | 17 | 9 |
| *M. guilliermondii* AGT1.3 | 37 | 39 | 38 | 38 | 35 | 54 | 54 | 53 | 69 | 68 | 100 | 53 | 54 | 55 | 53 | 53 | 53 | 31 | 39 | 19 | 32 | 40 | 42 | 22 | 41 | 41 | 38 | 17 | 10 |
| *L. elongisporus* AGT1 | 36 | 38 | 39 | 39 | 37 | 51 | 50 | 48 | 55 | 56 | 53 | 100 | 50 | 63 | 61 | 61 | 63 | 29 | 41 | 18 | 32 | 39 | 40 | 19 | 42 | 42 | 38 | 15 | 8 |
| *D. hansenii* AGT1 | 36 | 37 | 38 | 38 | 37 | 49 | 50 | 47 | 56 | 55 | 54 | 50 | 100 | 48 | 48 | 49 | 49 | 30 | 39 | 18 | 31 | 39 | 40 | 20 | 40 | 39 | 35 | 16 | 9 |
| *S. stipitis* MAL1 | 36 | 38 | 37 | 37 | 37 | 55 | 52 | 50 | 58 | 55 | 55 | 63 | 48 | 100 | 88 | 81 | 83 | 31 | 41 | 18 | 33 | 39 | 40 | 20 | 41 | 42 | 38 | 17 | 8 |
| *S. stipitis* MAL2 | 36 | 39 | 38 | 38 | 38 | 51 | 51 | 49 | 56 | 55 | 53 | 61 | 48 | 88 | 100 | 77 | 79 | 30 | 41 | 18 | 32 | 39 | 39 | 19 | 42 | 41 | 37 | 17 | 8 |
| *S. stipitis* MAL3 | 37 | 38 | 38 | 37 | 37 | 52 | 51 | 49 | 54 | 52 | 53 | 61 | 49 | 81 | 77 | 100 | 76 | 30 | 42 | 17 | 33 | 40 | 39 | 20 | 41 | 42 | 37 | 17 | 8 |
| *S. stipitis* MAL4 | 35 | 38 | 36 | 36 | 37 | 54 | 51 | 49 | 55 | 56 | 53 | 63 | 49 | 83 | 79 | 76 | 100 | 30 | 39 | 18 | 31 | 38 | 37 | 20 | 40 | 42 | 37 | 16 | 8 |
| *S. stipitis* MAL5 | 26 | 27 | 27 | 28 | 28 | 30 | 29 | 33 | 30 | 31 | 31 | 29 | 30 | 31 | 30 | 30 | 30 | 100 | 37 | 19 | 45 | 32 | 30 | 19 | 34 | 35 | 35 | 17 | 7 |
| *L. starkeyi* AGT1 | 32 | 36 | 37 | 37 | 35 | 41 | 41 | 45 | 43 | 41 | 39 | 41 | 39 | 41 | 41 | 42 | 39 | 37 | 100 | 19 | 38 | 45 | 43 | 20 | 47 | 47 | 41 | 17 | 8 |
| *L. starkeyi* AGT2.1 | 14 | 17 | 16 | 16 | 15 | 17 | 18 | 19 | 18 | 18 | 19 | 18 | 18 | 18 | 18 | 17 | 18 | 19 | 19 | 100 | 19 | 17 | 16 | 19 | 17 | 19 | 18 | 19 | 7 |
| *L. starkeyi* AGT2.2 | 29 | 29 | 29 | 30 | 28 | 31 | 31 | 35 | 34 | 34 | 32 | 32 | 31 | 33 | 32 | 33 | 31 | 45 | 38 | 19 | 100 | 34 | 33 | 23 | 36 | 35 | 38 | 18 | 10 |
| *L. starkeyi* AGT3 | 33 | 32 | 33 | 33 | 33 | 41 | 39 | 40 | 40 | 39 | 40 | 39 | 39 | 39 | 39 | 40 | 38 | 32 | 45 | 17 | 34 | 100 | 65 | 18 | 47 | 47 | 40 | 16 | 8 |
| *L. starkeyi* AGT4 | 34 | 35 | 36 | 36 | 36 | 42 | 40 | 41 | 43 | 42 | 42 | 40 | 40 | 40 | 39 | 39 | 37 | 30 | 43 | 16 | 33 | 65 | 100 | 18 | 43 | 44 | 38 | 15 | 7 |
| *L. starkeyi* AGT5 | 18 | 19 | 19 | 19 | 20 | 20 | 20 | 20 | 22 | 22 | 22 | 19 | 20 | 20 | 19 | 20 | 20 | 19 | 20 | 19 | 23 | 18 | 18 | 100 | 22 | 22 | 22 | 18 | 7 |
| *L. starkeyi* AGT6 | 34 | 36 | 36 | 37 | 34 | 41 | 41 | 41 | 42 | 42 | 41 | 42 | 40 | 41 | 42 | 41 | 40 | 34 | 47 | 17 | 36 | 47 | 43 | 22 | 100 | 51 | 44 | 17 | 9 |
| *A. oryzae* MalP | 33 | 36 | 37 | 37 | 35 | 41 | 41 | 42 | 42 | 41 | 41 | 42 | 39 | 42 | 41 | 42 | 42 | 35 | 47 | 19 | 35 | 47 | 44 | 22 | 51 | 100 | 43 | 16 | 10 |
| *B. adeninivorans* AGT1 | 32 | 32 | 31 | 31 | 31 | 38 | 37 | 38 | 40 | 38 | 38 | 38 | 35 | 38 | 37 | 37 | 37 | 35 | 41 | 18 | 38 | 40 | 38 | 22 | 44 | 43 | 100 | 17 | 8 |
| *S. pombe* Sut1 | 14 | 16 | 16 | 16 | 16 | 16 | 17 | 18 | 17 | 17 | 17 | 15 | 16 | 17 | 17 | 17 | 16 | 17 | 17 | 19 | 18 | 16 | 15 | 18 | 17 | 16 | 17 | 100 | 4 |
| *S. pombe* Ght3 | 9 | 8 | 7 | 7 | 8 | 9 | 9 | 9 | 10 | 9 | 10 | 8 | 9 | 8 | 8 | 8 | 8 | 7 | 8 | 7 | 10 | 8 | 7 | 7 | 9 | 10 | 8 | 4 | 100 |

**Table S5.** Primers for amplification of *Scheffersomyces stipitis* α-glucosidase genes of the *MAL* loci

| Gene | 5’-3’ | |
|---|---|---|
| | Forward | Reverse |
| MAL7 | ACCACTCTAGAAAGTAATAAATTCC | GAAAATTATCTTCGATTCATATTTAAGG |
| MAL8 | GATCTTCATGTATTCATAAATATTTGTACG | CCAATCCTTCTATGTATCTTCC |
| MAL9 | ATCTCATATTTCAGAATCAACTACTC | GGTTACTCTGGAGGACCCG |

**Table S6.** Primers for cloning the *Scheffersomyces stipitis* α-glucosidase genes to pURI3-Cter vector [1]

| Gene | 5’-3’ | |
|---|---|---|
| | Forward | Reverse |
| MAL7 | TAACTTTAAGAAGGAGATATACATATGACAATTGCTCGAGAATGGTGG | GCTATTAATGATGATGATGATGATGATCAACAATGTATAGACGACCTT |
| MAL8 | TAACTTTAAGAAGGAGATATACATATGACTGTTGCACACAAATGGTGG | TAACTTTAAGAAGGAGATATACATATGACTGTTGCACACAAATGGTGG |
| MAL9 | TAACTTTAAGAAGGAGATATACATATGACTAAAAGAATCTGGTGGAAG | GCTATTAATGATGATGATGATGATGGGACAACAAGTAGACCCGGGCT |

**Table S7.** Synteny between the *Ogataea polymorpha* NCYC 495 and *Ogataea parapolymorpha* DL-1 genomes according to Mycocosm data. Forward synteny between the chromosomes is shown on white background, reverse syntheny on grey background.

| Yeast | Chromosome No (length in Mbp) | | | | | | |
|---|---|---|---|---|---|---|---|
| *Ogataea polymorpha* NCYC 495 leu1.1 | 1 (1.54) | 2 (1.52) | 3 (1.36) | 4 (1.30) | 5 (1.27) | 6 (1.00) | 7 (0.99) |
| *Ogataea parapolymorpha* DL-1* | 7 (1.51) | 6 (1.51) | 5 (1.33) | 4 (1.29) | 3 (1.27) | 2 (0.99) | 1 (1.14) |

* Chromosome lengths were taken from [2]

**Table S8.** Hypothetical permease and α-glucosidase genes outside the *MAL* locus in *Ogataea polymorpha* and *Ogataea parapolymorpha* with their genomic neighbors indicated. Transcription direction is indicated by arrow.

| *Ogataea* strains (Chromosomes) | Genes and potential function of proteins | | |
|---|---|---|---|
| | copper transporter | α-glucosidase | gamma-tubulin |
| | → | ← | → |
| *O. polymorpha* NCYC 495 leu1.1 (Chr 7) | XP_018208523.1 | XP_018208522.1 | XP_018208521.1 |
| *O. parapolymorpha* DL-1 (Chr 1) | XP_013937410.1 | XP_013937411.1 | XP_013937412.1 |
| *Ogataea* strains (Chromosomes) | Genes and potential function of proteins | | |
| | clathrin coat assembly protein | α-glucoside permease | aminopeptidase |
| | ← | → | ← |
| *O. polymorpha* NCYC 495 leu1.1 (Chr 6) | XP_018209094.1 | XP_018209092.1 | XP_018209091.1 |
| *O. parapolymorpha* DL-1 (Chr 2) | XP_013936844.1 | XP_013936845.1 | XP_013936846.1 |

## References


1. Curiel, J. A.; de las Rivas, B.; Mancheño, J. M.; Muñoz, R. The pURI family of expression vectors: A versatile set of ligation independent cloning plasmids for producing recombinant His-fusion proteins. *Protein Expr. Purif.* **2011**, *76*, 44–53, doi:10.1016/j.pep.2010.10.013.
2. Ravin, N. V.; Eldarov, M. A.; Kadnikov, V. V.; Beletsky, A. V.; Schneider, J.; Mardanova, E. S.; Smekalova, E. M.; Zvereva, M. I.; Dontsova, O. A.; Mardanov, A. V.; Skryabin, K. G. Genome sequence and analysis of methylotrophic yeast *Hansenula polymorpha* DL1. *BMC Genomics* **2013**, *14*, 837.
